# Does the Slow-Growth, High-Mortality Hypothesis Apply Below Ground?

**DOI:** 10.1371/journal.pone.0161904

**Published:** 2016-08-29

**Authors:** James E. Hourston, Alison E. Bennett, Scott N. Johnson, Alan C. Gange

**Affiliations:** 1 School of Biological Sciences, Royal Holloway, University of London, Egham Hill, Egham, TW20 0EX, England; 2 The James Hutton Institute, Invergowrie, Dundee, DD2 5DA, Scotland; 3 Hawkesbury Institute for the Environment, Western Sydney University, Sydney, Australia; University of Oklahoma, UNITED STATES

## Abstract

Belowground tri-trophic study systems present a challenging environment in which to study plant-herbivore-natural enemy interactions. For this reason, belowground examples are rarely available for testing general ecological theories. To redress this imbalance, we present, for the first time, data on a belowground tri-trophic system to test the slow growth, high mortality hypothesis. We investigated whether the differing performance of entomopathogenic nematodes (EPNs) in controlling the common pest black vine weevil *Otiorhynchus sulcatus* could be linked to differently resistant cultivars of the red raspberry *Rubus idaeus*. The *O*. *sulcatus* larvae recovered from *R*. *idaeus* plants showed significantly slower growth and higher mortality on the Glen Rosa cultivar, relative to the more commercially favored Glen Ample cultivar creating a convenient system for testing this hypothesis. *Heterorhabditis megidis* was found to be less effective at controlling *O*. *sulcatus* than *Steinernema kraussei*, but conformed to the hypothesis. However, *S*. *kraussei* maintained high levels of *O*. *sulcatus* mortality regardless of how larval growth was influenced by *R*. *idaeus* cultivar. We link this to direct effects that *S*. *kraussei* had on reducing *O*. *sulcatus* larval mass, indicating potential sub-lethal effects of *S*. *kraussei*, which the slow-growth, high-mortality hypothesis does not account for. Possible origins of these sub-lethal effects of EPN infection and how they may impact on a hypothesis designed and tested with aboveground predator and parasitoid systems are discussed.

## Introduction

The black vine weevil, *Otiorhynchus sulcatus* Fabricius (Curculionidae) causes significant damage to a range of silvicultural and horticultural crops throughout the world’s temperate regions [1]. Adult *O*. *sulcatus* feed on the foliage of a huge range of plants, inflicting relatively minor damage when compared to the root feeding larvae, which can result in reduced plant growth and if an infestation is severe, the death of a host plant [2]. Conventional control of *O*. *sulcatus* is achieved using soil drench treatments of chemical pesticides. Until very recently the most commonly used treatment for an *O*. *sulcatus* infestation was the neonicotinoid; Imidacloprid, which has been temporarily withdrawn from use in the EU since 2014 due to non-target effects on bees. Future strategies to control *O*. *sulcatus* would be wise to therefore consider pesticide free alternatives as part of an integrated approach to pest management [3]. An alternative method of control popular for the treatment of plants that may be at risk of, or already under *O*. *sulcatus* attack, is entomopathogenic nematodes (EPNs). These have been shown in many studies to be effective in reducing both the performance and increasing mortality of *O*. *sulcatus* [4–6].

One of the primary plant hosts of *O*. *sulcatus* which is of major economic importance is the red raspberry, *Rubus idaeus* L.(Rosaceae), with over 13.8 thousand tons produced in 2013, worth $135.7 million (GPB/USD 1.52 on 23/11/2015) to the UK economy [7] and in the USA, in 2014 a production of 97 thousand tons worth $499.3 million, by Washington state, California and Oregon alone [8]. The production of *R*. *idaeus* is, in the UK, almost entirely under the protection of plastic tunnels which can raise temperatures by around 4°C compared to the surrounding field conditions and results in greatly increased growth [9]. However these conditions are also very favorable for *O*. *sulcatus* performance with the insects consuming more *R*. *idaeus* biomass, completing their life cycles faster and with adults being more fecund [9]. Two cultivars that have been studied previously with respect to their tolerance to *O*. *sulcatus* attack are Glen Ample and Glen Rosa [10]. Despite being closely related autumn mid-season fruiters these cultivars differ in their usage, with Glen Ample being a major commercial variety, due to fruit size and quality, and Glen Rosa being more popular on the amateur market due to its better tolerance to pests and diseases [11,12].

This study utilized these two cultivars of *R*. *idaeus*, demonstrated to be more (Glen Rosa) and less (Glen Ample) resistant to *O*. *sulcatus* [10]. Comparing two differently resistant cultivars is likely to result in different growth rates in *O*. *sulcatus* and which enables the testing of Feeny’s (1976) [13] slow growth, high mortality hypothesis when these two *O*. *sulcatus* populations are exposed to natural enemies. This hypothesis predicts that when plant traits impose a cost to the fitness of larvae, increasing developmental time, either via sub lethal effects via antixenosis, antibiosis or the reallocation of resources away from sites of herbivory, that herbivores will be vulnerable for longer to predation. Thus counteracting the effects of reduced host plant nutritional quality that might result in herbivores compensating by increasing consumption of plant tissues. The inclusion of natural enemies in order to truly understand plant-herbivore interactions has long been championed in the field of tri-tropic interactions [14] and continues to be further explored in light of new evidence [15]. *Otiorhynchus sulcatus* is a good model for testing the hypothesis that resistant cultivars and EPNs may be combined as a technique to suppress root herbivore populations as *O*. *sulcatus* has a history, particularly in the horticultural sector, of being treated with a range of EPNs [16]. This alongside evidence from field studies reporting that heavy infestations of *O*. *sulcatus* reduced yield by 39% and 66% in Glen Rosa and Glen Ample [10], respectively, provides a framework for a convenient multi-trophic system in which to study potential interactions.

The two EPN species incorporated into the experiment are both widely recommended and commercially available specifically for use against *O*. *sulcatus* [4]. *Steinernema kraussei* Steiner is cold tolerant, active at <10°C, whereas *Heterorhabditis megidis* Poinar, Jackson & Klein is active at >10°C, both are known to alter their dispersal and taxis depending on the substrate they are in and possess different bacterial endosymbiont communities [5,17,18].

To assess how the different EPN treatments influenced *O*. *sulcatus* mortality and performance we proposed two hypotheses. First that EPN treatments would decrease *O*. *sulcatus* abundance, as one would expect from a tried and tested biological control agent. Following on from this we predicted that the presence of the more resistant host, Glen Rosa, would, in combination with EPNs, result in lower larval mass and higher levels of *O*. *sulcatus* control. This hypothesis was based on Feeny's, (1976) hypothesis that slow growth leads to high mortality from natural enemies. So, we would expect that on a more resistant host, *O*. *sulcatus* would have a slower growth and consequently be more susceptible to EPN infection. Plant biomass was calculated at the end of the experiment to quantify the plant response to EPN treatments. We hypothesized that EPN treatments on plants infested with *O*. *sulcatus* would promote an increase in plant biomass as the plants should be suffering less herbivore damage. The proportion of root biomass to shoot biomass was used as an indirect measure of changes in carbon allocation in response to herbivory. We predicted that the EPN treatments and subsequent abundance of *O*. *sulcatus* would influence the proportion of root and shoot biomass in *R*. *idaeus* cultivars, potentially allowing a recovery in root biomass following successful *O*. *sulcatus* control.

## Materials and Methods

Rootstock from existing *R*. *idaeus* plants of two cultivars; Glen Ample and Glen Rosa was sourced from The James Hutton Institute’s (Dundee, UK) breeding stock. The rootstock was washed with a 4% sodium hypochlorite solution, rinsed with water then planted in twice autoclaved (with a 12hr gap between autoclave runs) compost (Keith Singleton sterilized loam, Nethertown, UK) and grown on under-heated benches in a controlled greenhouse environment (16:8 days at 18˚C). Four weeks after this, plants were transplanted into 1.8L pots containing 1.6L of a homogenized, twice autoclaved 1:1 soil (Keith Singleton sterilized loam, Nethertown, UK) and sand mix. Compost was autoclaved in batches to control for the possibility of there being any nematodes living in the compost which could interact with added treatments [19]. Plants from each cultivar were equally and randomly distributed between the 4 treatments giving a replication of 26 plants for each treatment. To account for any possible environmental heterogeneity within the glasshouse the plants were then incorporated into blocks each equally representing individuals from each treatment in a randomized order to adhere to a randomized block design. Two weeks after the plants were transplanted, and before any herbivore or EPN treatments were added, plant height was recorded in order to be used later as a random effect in statistical models to account for the initial variation in height between plants.

**Fig 1:**
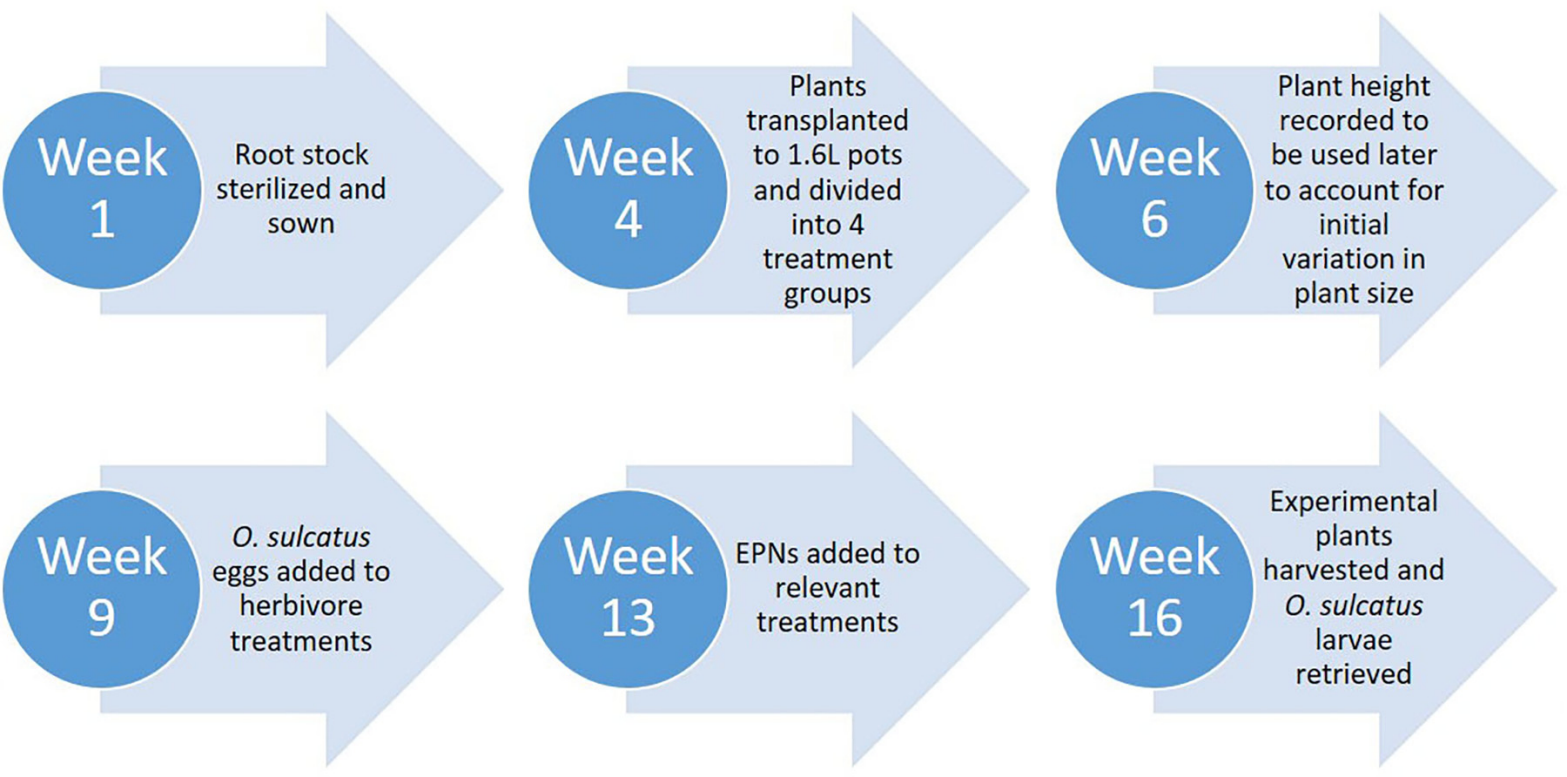
The timing of key stages to the experimental setup and execution.

Five weeks after *R*. *idaeus* were transplanted into individual pots, 40 *O*. *sulcatus* eggs were added into a 10mm indent in the soil surface, 20mm away from the stem of each plant. This egg density was selected to simulate arrival of a gravid adult feeding on plants for several weeks [10]. Four weeks after plants were infested with *O*. *sulcatus*, EPNs were added to plants, with control plants remaining untreated. Three weeks after nematodes were added, the plants were harvested and *O*. *sulcatus* larvae were retrieved, counted and fresh mass taken. A subset of half the plants were then freeze dried to ascertain dry mass (**Fig 1**). *Otiorhynchus sulcatus* eggs were taken from a culture of adults maintained at 18˚C with a 16:8 light: dark cycle at The James Hutton Institute, Dundee. The EPNs used in the experiment were purchased from commercial suppliers and were advertised as being a specific line to control for *O*. *sulcatus*. *S*. *kraussei* (Becker and Underwood®, Littlehampton, UK) and *H*. *megidis* (Biobest®, Milton Bridge, UK). They were both added to plants as separate treatments at their recommended dosages. When formulated to the commercially recommended dosages this results in 7735 ± 531 *S*. *kraussei* added per pot and approximately 12275 ± 780 *H*. *megidis* added to each pot according to the product formulation guidelines.

This created a 2 × 4 factorial experiment which was conducted under controlled conditions (16:8 days at 18˚C). Two *R*. *idaeus* cultivars (Glen Ample and Glen Rosa) were subdivided into one of four treatments. A control treatment, where neither *O*. *sulcatus* or EPNs were added, an *O*. *sulcatus* ‘only’ treatment where the herbivore was observed in the absence of EPNs and two treatments in which, in addition to *O*. *sulcatus*, either *S*. *kraussei* or *H*. *megidis* were added to plants.

### Statistical analysis

The mean mass and abundance of *O*. *sulcatus* larvae on each plant was analyzed using generalized linear mixed models (GLMMs) incorporating Gaussian and Poisson errors respectively. These response variables were tested against the cultivar and EPN treatment and the interactions between the two. Experimental block and autoclave batch were included as random effects. The biomass data taken from the dry mass of *R*. *idaeus* plants was also analyzed using a GLMM incorporating Gamma errors with a log link, using nematode treatment, *O*. *sulcatus* abundance and mean *O*. *sulcatus* mass as explanatory variables with experimental block included as a random effect. All analyses were carried out using R3.2.3 "Wooden Christmas-Tree" [20] using the lmer4 [21] and car [22] packages for GLMMs. Models were simplified where appropriate using AIC, with the best fitting minimal models reported. Pairwise comparisons were conducted using the R package phia [23].

## Results

### Insect herbivore performance

**Fig 2:**
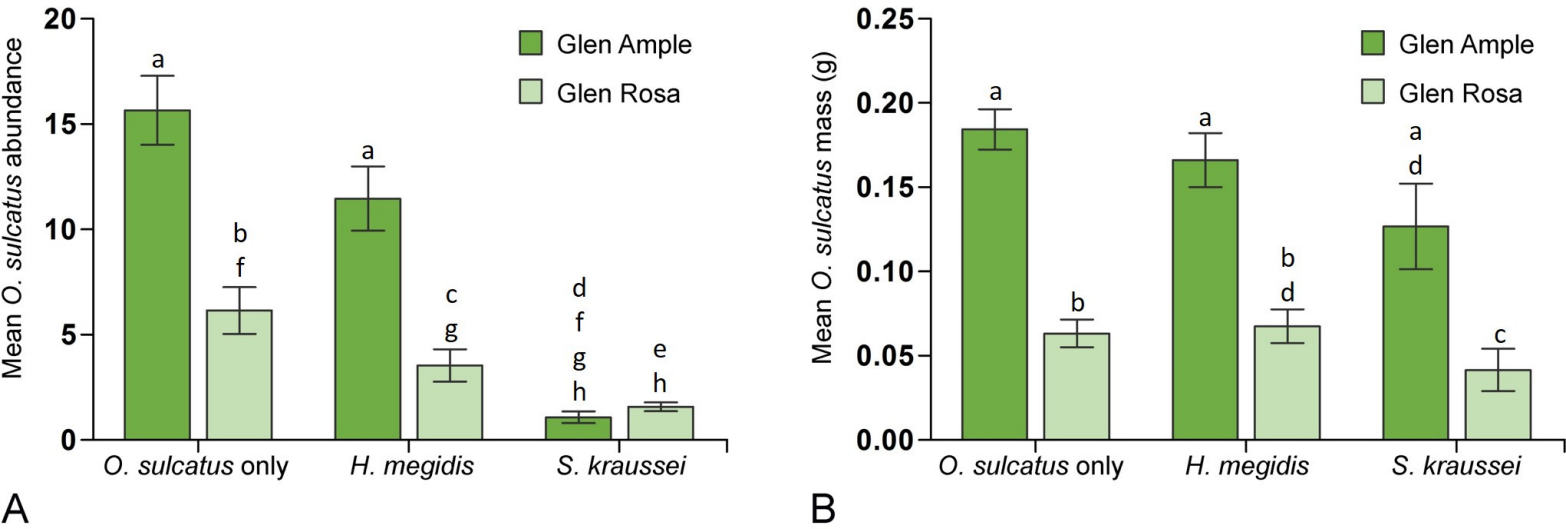
**A** Mean *O*. *sulcatus* abundance per plant across different *R*. *idaeus* cultivar and EPN treatments. **B** The mean mass of *O*. *sulcatus* larvae per plant recovered from different *R*. *idaeus* cultivars and EPN treatments. Statistical differences between treatments are indicated by different letters above the bars.

The abundance of *O*. *sulcatus* recovered at the end of the experiment was affected by both the *R*. *idaeus* cultivar and the EPN treatment added to plants (**Table 1**). In both cultivars the addition of *S*. *kraussei* resulted in lower abundance when compared to control treatments and treatments where *H*. *megidis* were added (**Fig 2A**). In addition to the observed differences between cultivars on *O*. *sulcatus* abundance, the mass of recovered larvae was found to be lower (χ^2^ = 74.11, d.f. 1, P<0.001) in Glen Rosa when compared to Glen Ample (**Fig 2B**). The mass of larvae was also lower on *S*. *kraussei* treated plants when compared to *O*. *sulcatus* only treatments (χ^2^ = 7.39, d.f. 1, P<0.05).

**Table 1:** Summary table of post hoc contrasts carried out on *O*. *sulcatus* abundance to describe the interactions between *R*. *idaeus* cultivar and mean *O*. *sulcatus* mass and *R*. *idaeus* cultivar and EPN treatment.

Contrast: Category	*df*	χ^2^ value	*P*-value
**Cultivar: Mean *O*. *sulcatus* mass**	1	4.46	<0.05
***O*. *sulcatus* only -*H*. *megidis*: Glen Ample**	1	1.54	0.21
***O*. *sulcatus* only -*S*. *kraussei*: Glen Ample**	1	60.37	<0.001
***H*. *megidis-S*. *kraussei*: Glen Ample**	1	45.89	<0.001
***O*. *sulcatus* only -*H*. *megidis*: Glen Rosa**	1	5.83	<0.05
***O*. *sulcatus* only -*S*. *kraussei*: Glen Rosa**	1	26.07	<0.001
***H*. *megidis-S*. *kraussei*: Glen Rosa**	1	9.06	<0.001
**Glen Ample-Glen Rosa: *O*. *sulcatus* only**	1	14.66	<0.001
**Glen Ample-Glen Rosa: *H*. *megidis***	1	20.62	<0.001
**Glen Ample-Glen Rosa: *S*. *kraussei***	1	0.65	0.41

**Fig 3:**
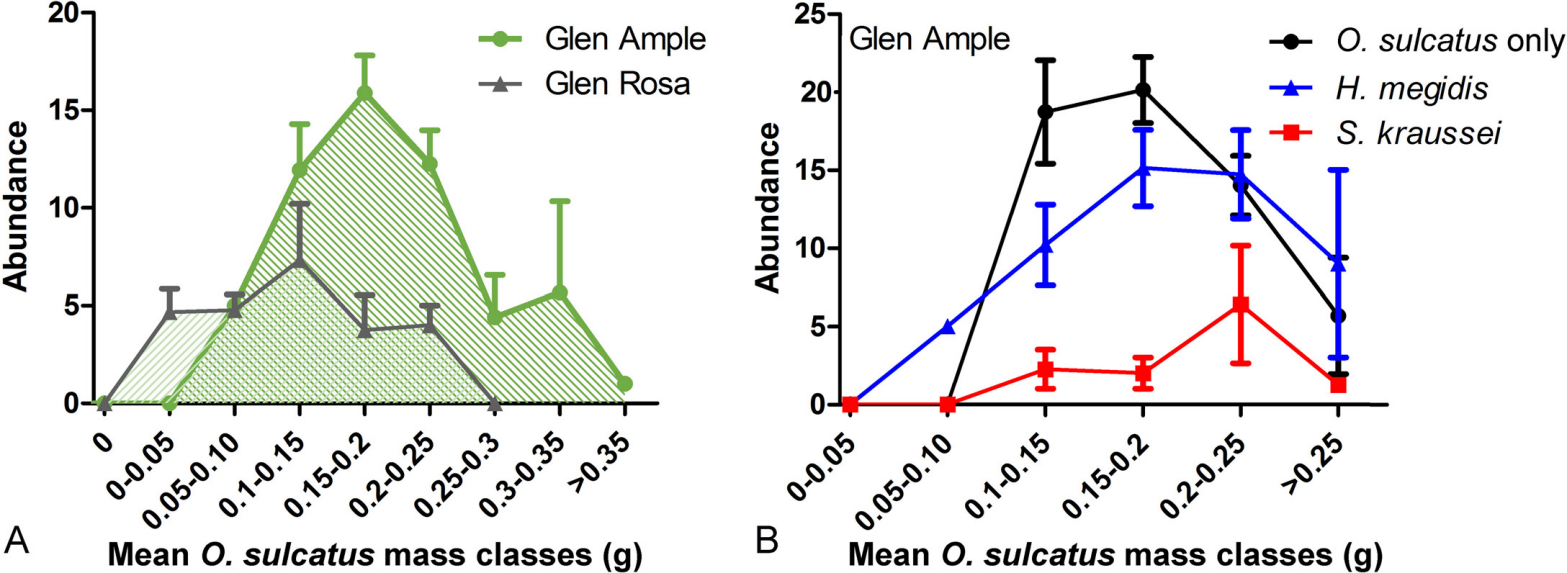
**A** The relationship between *O*. *sulcatus* abundance and mean mass across two *R*. *idaeus* cultivars. **B** The relationship between *O*. *sulcatus* abundance and mean mass within Glen Ample across EPN and *O*. *sulcatus* only treatments.

Differences observable between the abundance of *O*. *sulcatus* on the two cultivars were entirely driven by an interaction (**Table 1**) between the mean mass of *O*. *sulcatus* and the cultivar of *R*. *idaeus*. Larvae on Glen Ample had a greater mass and suffered lower mortality, while those on Glen Rosa had a low masses and suffered greater levels of mortality (**Fig 3A**). There was an impact of EPNs on this relationship within Glen Ample with *S*. *kraussei* exhibiting a different relationship as observed in the *H*. *megidis* and *O*. *sulcatus* only treatments (χ^2^ = 4.54, d.f. 1, P<0.05). While the *H*. *megidis* and *O*. *sulcatus* only treatments followed the general trend observed for Glen Ample in **Fig 3A**, *S*. *kraussei* caused a reduction (χ^2^ = 7.28, d.f. 1, P<0.05) in *O*. *sulcatus* mass as well abundance (**Fig 3B**). Within Glen Rosa, there was not found to be a significant difference between the *O*. *sulcatus* only, and EPN treatments, so far as their impact on the relationship between *O*. *sulcatus* abundance and mean body mass was concerned.

### Plant biomass

**Fig 4:**
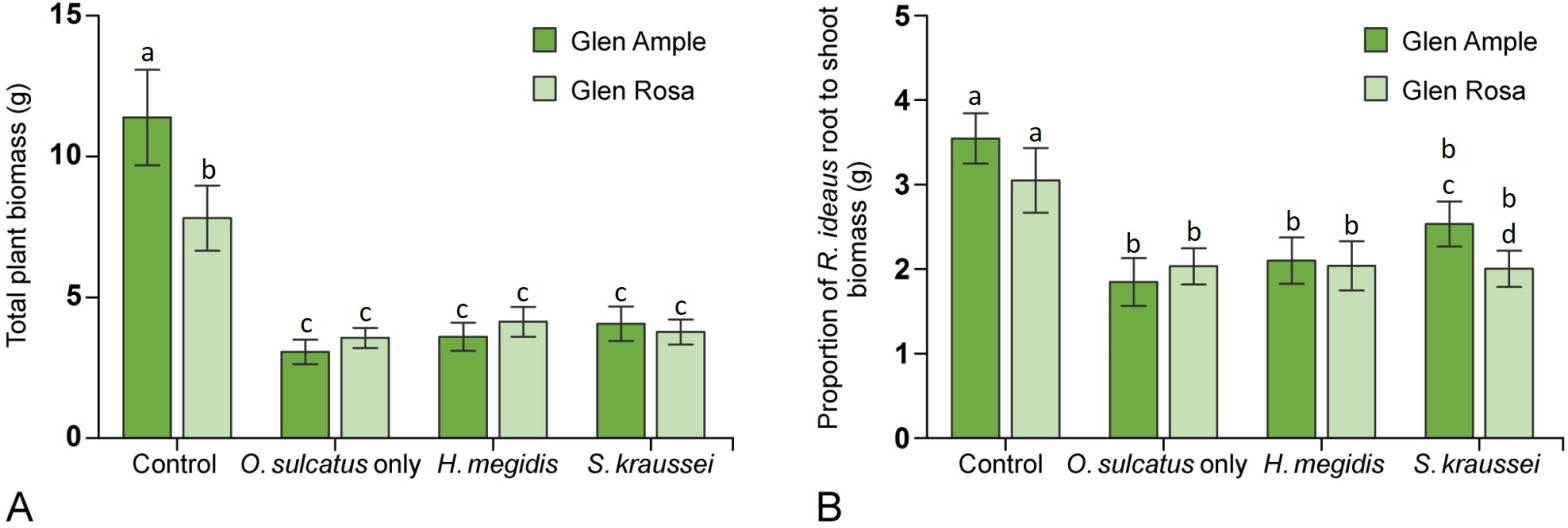
**A** The total biomass of *R*. *idaeus* of different cultivars across EPN treatments. **B** The proportion of root to shoot biomass measured at the end of the experiment separated by *R*. *idaeus* cultivar and EPN treatment. Statistical differences between treatments are indicated by different letters above the bars.

The total plant biomass calculated at the end of the experiment showed that the two *R*. *idaeus* cultivars performed differently when herbivores were present (**Fig 4A**). A decrease in biomass was particularly clear in Glen Ample, where average biomass fell by >50% when herbivores were added (χ^2^ = 18.48, d.f. 1, P<0.001) a pattern that was not reflected in Glen Rosa’s total biomass once variation in initial plant height was taken into account. In the herbivore free control treatment, the two cultivars had a different mean total biomass with Glen Ample having a significantly higher biomass than Glen Rosa (χ^2^ = 5.07, d.f. 1, P<0.05).

The distribution of *R*. *idaeus* biomass between the above and belowground portions of the plant also appeared to have been disrupted by the presence of *O*. *sulcatus* (**Fig 4B**). The proportion of root to shoot biomass showed a general trend for higher root biomass relative to shoot biomass in the control treatment. In the *O*. *sulcatus* only, and *H*. *megidis* treatments both cultivars showed very similar trends in how biomass was distributed. The only exception was in plants treated with *S*. *kraussei*, where a higher proportion of root biomass relative to shoot biomass was recorded in Glen Rosa than in Glen Ample (χ^2^ = 15.37, d.f. 1, P<0.001).

## Discussion

The comparison of two commercially available EPN species showed that *S*. *kraussei* was more effective at controlling *O*. *sulcatus* than *H*. *megidis* in both cultivars of *R*. *idaeus*, with the abundance and growth of *O*. *sulcatus* being substantially reduced. Both these EPN species are considered to be capable of cruise foraging, meaning they actively seek out hosts in the soil [17,24]. The experiment was held at a constant 18°C meaning both species were operating within their optimal temperature range. Their contrasting performance could hence be due to other differences in behavior and biology. There have been several studies that show soil media or substrate can have a significant effect on the dispersal behavior of EPNs, with different species showing greater taxis towards hosts in different media [5,17]. This could explain some of the variation between these species and consequently results may not be the same in the field. This said, *S*. *kraussei* has a lower cold tolerance (4°C) when compared to *H*. *megidis* (10°C) making it a better choice when treating plants at the beginning or end of a growing season [4]. This is ideal for the protection of both Glen Ample and Glen Rosa, as these are both mid-season fruiting varieties, and the beginning of the season represents a period of critical growth, prior to flowering [12]. The poorer performance of *H*. *megidis* could be due to a better *O*. *sulcatus* immune response. Possibly resulting in successful encapsulation of EPNs, or resistance to the associated symbiotic bacteria, *Photorhabdus spp*., which normally causes death by septicemia, different to the *Xenorhabdus spp*. associated with *S*. *kraussei* [25].

*Otiorhynchus sulcatus* performed significantly better on Glen Ample plants than on Glen Rosa as shown in their larval mass, and this is supported by previous studies which found Glen Ample to be a cultivar less resistant to *O*. *sulcatus* when compared to other *R*. *idaeus* cultivars [10,26]. This is likely due to the different traits bred into these two cultivars. Glen Ample is a more popular variety as it produces a higher yield of larger, sweeter fruit and is favored commercially. Glen Rosa however is more tolerant to pests and diseases. It has been bred to have an A_10_ resistance gene which confers resistance to the large raspberry aphid, *Amphorophora idaei* Börner, but has smaller fruit and typically produces smaller yields when compared to Glen Ample [12].

The testing of Feeny’s (1976) slow growth, high mortality hypothesis using the comparison between *O*. *sulcatus* performance on Glen Ample and Gen Rosa would appear to be highly appropriate as exactly this relationship of *O*. *sulcatus* growth and mortality was observed between the two cultivars. It is apparent that *H*. *megidis* fits to this model, as when *O*. *sulcatus* mass was low on Glen Rosa, *O*. *sulcatus* abundance fell accordingly. However, the same degree of conformity to Feeny’s hypothesis was not observed in *S*. *kraussei*, where abundance of *O*. *sulcatus* was found to be low regardless of changes in *O*. *sulcatus* mass. There may be several reasons for this nonconformity, firstly the mass of *O*. *sulcatus* larvae recovered in the experiment was not just affected by the difference in cultivar. The addition of *S*. *kraussei* also appeared to directly decrease larval mass in *O*. *sulcatus*. This might suggest that there are potential sub-lethal effects being observed in the surviving larvae. *O*. *sulcatus* may be infected by EPNs and this stress may impact on feeding rates and larval development [27] but not result in death. The level of tolerance to EPNs is known to vary greatly, with some insect immune systems able to encapsulate and withstand up to 20 EPNs before the insect’s death [28]. Secondly as Feeny’s hypothesis was based around more classical aboveground predator/parasitoid systems it may be that the more complex communal life strategy employed by EPNs may be less appropriate. There is even evidence that EPNs can interact directly with plant defense chemistry, inducing systemic resistance in plants [29].

Perhaps greater than the interactions that occur directly between the plant and EPNs is the influence of the staggeringly complex soil microbial community. The soil microbial community, when studied in model plants, has been found to be extremely large, taxonomically diverse and specific to certain soil types [30,31]. Indeed the differences in soil structure and composition that have been identified as being a determining factor in EPN efficacy [5,17] also have a strong effect in determining the soil microbial community composition [32]. There are many examples of soil microbes interacting with plants to bolster plant defenses against pests and pathogens [33] but also, soil based entomopathogenic microbes can provide competition for EPNs which can lead to their competitive exclusion from insect hosts [34]. It is therefore likely that in such a complicated system as soil, the slow-growth, high-mortality hypothesis is unlikely to prove a comprehensive explanation for the myriad interacting organisms and their impacts in plant/herbivore/natural enemy interactions.

The difference in total plant biomass observed between the *R*. *idaeus* cultivars in the control treatment followed what would be expected from the traits bred into these lines. The more vigorous growth more typically associated with Glen Ample [12] would be expected to lead to greater average biomass than in Glen Rosa. The lower biomass observed, particularly in Glen Ample, when *O*. *sulcatus* was present fitted well with field observations [10].

Host plants have an arsenal of different defences that they can deploy against herbivores such as antixenosis, antibiosis and tolerance [35]. Even differing plant traits such as variation in nutritional value of plant tissues between two cultivars or species can reduce slower growth in herbivores resulting in higher natural enemy related mortality [36]. Differences in the nutritional chemistry of the roots of the two cultivars may play a role in differing *O*. *sulcatus* performance. *O*. *sulcatus* larval abundance has been shown to be positively correlated with levels of nitrogen and magnesium and negatively so with respects to iron, but this was not found to be significantly different between Glen Ample and Glen Rosa cultivars [26]. The A_10_ resistance gene bred into Glen Rosa after being isolated from *R*. *occidentalis* L. is thought to be effective against aphids through both antixenosis and antibiosis [37]. It has been suggested in other studies that the presence of this gene in Glen Rosa may be conferring resistance against *O*. *sulcatus* [11,26]. This differing plant trait bred into the two cultivars may well explain the observed reduction in larval performance on Glen Rosa. In this experiment, herbivores are not given a choice of host plants and *O*. *sulcatus* are known to readily consume both Glen Ample and Glen Rosa in the absence of choice [11] and so the effects observed are not driven by antixenosis.

There are many different ways by which antibiosis can be affected, either through abiotic, for example via increased plant nutrition [38], or biotic, via association with beneficial microbes [33], means. Conducting controlled glasshouse experiments minimises the impact of many of these factors and so effects that are observed are most likely as a consequence of plant traits. When a plant is damaged by a feeding herbivore, constitutive and inducible defences are activated which usually involve the increased production of secondary metabolites. Compounds such as alkaloids, glucosinolates, terpenoids, and phenolics can have a variety of different, lethal and sub lethal effects on plant herbivores [39]. Phenolics for example have been identified as likely to act as an antifeedant in *Ribes nigrum* L. decreasing *O*. *sulcatus* performance [40,41]. Higher concentrations of phenolic compounds or similar secondary metabolites in Glen Rosa, relative to Glen Ample, could explain the reduction in the performance of *O*. *sulcatus* larvae when EPNs were not present. Such a plant trait would be a classic example of how plant defence chemistry can extend the most vulnerable period of the insect life cycle, exposing herbivores to predation.

Induced defences often include an increase in the concentration of volatile organic compounds (VOCs) present in plant tissues which can act in many different ways, either as a direct toxin or a feeding deterrent [42]. These VOCs are exuded by the plant both above and belowground and in some cases this is used by additional herbivores to locate and identify an already damaged plant, but this can also act as an attractant for natural enemies that can come to the aid of the attacked plant. The EPN, *H*. *megidis* has been shown to be attracted to volatile emissions from plants that have been attacked by herbivores [43]. It may be that the decreased abundance of *O*. *sulcatus* on Glen Rosa could be attributed to greater concentrations of VOCs released from root tissues at sites of tissue damage which could be attracting EPNs towards to their host’s location. Examining the interplay between plants, herbivores, natural enemies and how VOC emissions tie them all together is a growing field that may in future provide new territory for breeding in new types of pest resistant traits [44].

There are various definitions and means of measuring plant tolerance to herbivory but one that can be assessed in this experiment is the difference in fitness between damaged and undamaged plants compared between cultivars [45]. If the biomass collected at the end of the experiment could be considered an indication of *R*. *idaeus* fitness then it is clear that compared to Glen Ample, Glen Rosa is exhibiting tolerance to *O*. *sulcatus* herbivory. With Glen Ample suffering a large decrease in biomass as a consequence of the presence of *O*. *sulcatus* but Glen Rosa maintaining a similar biomass. There is evidence of compensation for lost biomass in Glen Rosa, a classic tolerance mechanism. It is however hard to determine if this may come at a cost to fitness as the plants were not grown for long enough to assess seed production. The ability of a plant to shift carbon stores from roots to shoots, thus changing the distribution of biomass, is another recognised indication that a plant tolerance mechanism is occurring [45]. There were no clear indications that root to shoot biomass demonstrated this tolerance mechanism as there was only a trend of decreased root biomass relative to shoot biomass when *O*. *sulcatus* were present. This general trend was reversed however in the *S*. *kraussei* treated plants, where the successful reduction in *O*. *sulcatus* abundance appears to restore a more vigorous root growth in Glen Rosa. The general pattern of decreased root biomass relative to shoot biomass under root herbivory is not unsurprising. A pattern of resource reallocation away from insect herbivory has been observed in previous studies, suggesting this may be an established plant defense strategy [46,47].

### Conclusions

*S*. *kraussei* performed best out of the two EPN species tested, possibly due to better suitability to the soil substrate, a key factor influencing EPN efficacy. Another key factor that commonly affecting EPN efficacy [30,31], temperature, was discounted as having an effect as both species tested were within their optimum temperature range.

Differences between the two raspberry cultivars tested were likely due to herbivore resistance bred into the Glen Rosa cultivar. The presence of high concentrations of phenolic compounds have been known to affect *O*. *sulcatus* in other soft fruit crops [40,41]. Despite its relative susceptibility to *O*. *sulcatus*, Glen Ample remains the commercial favourite due to its high yield of large fruits. However, as pesticides that are effective for controlling *O*. *sulcatus* are withdrawn from the market, over safety and environmental concerns, more resistant plants may increasingly become attractive as part of an integrated crop management approach.

Lack of conformity by the EPN *S*. *kraussei* to the slow growth, high mortality hypothesis could be explained by lower *O*. *sulcatus* larval masses in *S*. *kraussei* treatments which indicates sub-lethal effects of exposure to this EPN. This hypothesis was not originally devised with EPNs in mind and has been primarily been tested with data from predator and parasitoid natural enemies [48,49] and may not, for this reason, sufficiently explain such interactions especially in complex soil ecosystems.
